# Before hormones: early sex-specific mechanisms shaping nervous system development, function, and postnatal disease susceptibility

**DOI:** 10.3389/fnbeh.2026.1888207

**Published:** 2026-08-28

**Authors:** Maria A. Tsiarli, Erica N. Larschan

**Affiliations:** 1 Department of Molecular Biology, Cellular Biology and Biochemistry, Brown University, Providence, RI, United States; 2 Carney Institute for Brain Science, Brown University, Providence, RI, United States

**Keywords:** brain development, neurogenesis, dosage compensation, female, male, sex, stem cells, transcriptional regulation

## Abstract

Brain development requires precise spatiotemporal regulation of gene expression and molecular processes to establish healthy and functional neural circuits. Traditionally, sex differences in brain development have been attributed primarily to gonadal differentiation and the subsequent actions of sex hormones on developmental systems during fetal life. However, these observations alone cannot fully explain the spectrum of sex biases seen in diverse neurodevelopmental, neuropsychiatric, and neurological disorders. In this mini review, we discuss the current evidence supporting the early embryonic origins of sex differences in brain development, with an emphasis on sex chromosome-driven transcriptional regulation, X chromosome inactivation dynamics, and sex-biased gene expression, which precede hormone-dependent developmental signaling. We present recent advances using omics technologies and computational approaches that have revealed widespread sex-biased differences in gene expression, chromatin regulation, dosage compensation, alternative splicing, and transcriptional activity during preimplantation and early embryonic development. These results are derived from available human datasets, but also include data from experimental models, including *Drosophila melanogaster,* and elaborate on the translational application of these findings in humans during normal neurodevelopment and in disease states. Collectively, these findings support a model in which sex-specific features of brain development emerge from the earliest stages of embryogenesis and continue to shape brain structure and function throughout life. Furthermore, they emphasize the necessity of including sex as a variable in research and medicine.

## 1 Introduction

Traditionally, sex differences in brain development have primarily been attributed to gonadal differentiation and the subsequent actions of sex hormones during fetal life. While sex steroid hormones exert powerful and transformational control over brain sexual differentiation and neural circuit development, there are still unanswered questions regarding the observed sex dimorphism in many brain-related disorders, from neurodevelopmental disorders to cognitive decline and even brain cancer. Additionally, there are significant morphogenetic events that take place during embryogenesis well before gonad formation and sex-specific gonad activation, including neurulation and nervous system development. However, the majority of reviews discussing sex biases in nervous system development do not examine these initial and integral steps or the mechanisms that guide them, leaving a major gap in our understanding of how sex shapes neural circuit formation and function.

Sex is a binary trait defined by the sex chromosomes. Thus, sex differences in heterogametic species, including humans, arise from the different combination of sex chromosomes X and Y, XX in typical females and XY in typical males. The mammalian X chromosome is remarkable for its gene composition, as it contains an enrichment of brain-related, reproduction-specific genes. Interestingly, in humans, it shows an enrichment of male-specific genes (Graves et al., 2002; Leitão et al., 2022; Lercher et al., 2003). Due to the imbalance arising in the gene complement of the sex chromosomes between the two sexes, dosage-compensation mechanisms have evolved to restore the balance between autosomal and sex gene expression (Cecalev et al., 2024). In humans, X dosage balancing takes place through the random transcriptional inactivation of one of the female X chromosomes, through a process guided by the long non-coding RNA (lnRNA), XIST (X-inactive specific transcript). Nonetheless, in contrast to other mammals, both X chromosome expressions are active in human female preimplantation embryos, and a gradual dampening of both X chromosome expression takes place (Petropoulos et al., 2016) in a process that also involves another lnRNA, XACT (X-active coating transcript), which coats the active X chromosome in human pluripotent cells (Casanova et al., 2019; Vallot et al., 2013). Therefore, from the moment of conception, the complement of sex chromosomes carried by the embryo dictates the molecular steps that will occur as morphogenesis and nervous system formation take place.

Sex-based differences can be seen in the differential effects that the womb environment/maternal factors can exert on the developing embryo’s nervous system. In a recent study, Wang et al. (2026) showed that maternal exposure to high levels of estradiol resulted in differential responses in gene expression between male and female fetal regions and in the proliferation and differentiation dynamics of cortical neural progenitor cells in male mice. This study was performed at embryonic day (E) 18.5, a developmental stage that falls within the critical period of hormone-dependent sexual differentiation of the mouse brain and is representative of the stages examined in many studies evaluating sex-based differences. By E18.5, neural progenitors have largely transitioned toward gliogenesis; therefore, the reported sex-based differences primarily reflect late-stage neural progenitor cells (NPCs). Nonetheless, it is critical to investigate sex-based differences during earlier stages of embryogenesis and neurogenesis, when early NPCs undergo extensive molecular and cellular changes that shape subsequent nervous system formation. Thus, it is critical for the field to investigate and understand sex-based differences during the early stages of embryogenesis and neurogenesis.

In this mini review, we focus on the following question: *When do sex differences in neurodevelopment first emerge, and which molecular mechanisms establish them before hormone-regulated differentiation*? We discuss the current evidence supporting the early embryonic origins of sex differences in nervous system and forebrain development, with an emphasis on sex chromosome-driven transcriptional regulation, X chromosome inactivation dynamics, sex-biased gene expression, and hormone-dependent developmental signaling. The results discussed include data from neural stem cells and the entire human brain. We further examine how these mechanisms contribute to neural development and disease vulnerability across the lifespan. Collectively, these findings support a model in which sex-specific features of brain development emerge from the earliest stages of embryogenesis and continue to shape brain structure and function throughout life.

## 2 Sex-differences timing: how early do they take place?

The majority of studies in the field of developmental neurobiology have focused on sex differences related to sex hormones. Nonetheless, with new data and technologies emerging, it is becoming clear that sex-specific genetically encoded differences are present from the very early stages of embryonic development owing to differential layers of regulatory mechanisms, including the control of transcription and splicing of genes encoded on sex chromosomes and their interplay with autosomes (Richardson et al., 2023; Bermejo-Alvarez et al., 2011).

### 2.1 Timing of sex-biased differences following zygote formation

Activation of genomic transcription on the two sex chromosomes occurs at different times and at different levels. Single-cell RNA-seq during early human embryogenesis revealed that transcription of X-linked genes is activated immediately following embryonic genome activation in both male and female embryos. Female embryos show higher X-linked gene expression compared to males at the 8-cell and morula stages, while autosomal gene expression becomes more evident as the blastocyst stage progresses (Zhou et al., 2019). Y chromosome-specific expression was detected at the 8-cell stage, with expression of genes *RPS4Y1* and *DDX3Y* surpassing the expression levels of a group of other 29 Y-specific genes. Zhou et al. (2019) noted high *RPS4Y1* expression levels during the initial stages of Y chromosome activation, following zygotic genome activation, which can be used as a sex-specific identifier for cleavage-stage human embryos. Furthermore, during the early stages of *in vitro* neural differentiation in human embryonic stem cells, *RPS4Y1* was found to be the only Y-linked gene with maximum expression (Pottmeier et al., 2024). A very interesting study that employed computational analyses of previously published single-cell RNA-sequencing datasets, from human 8-cell to late blastocyst stages and mouse 2-cell to early blastocyst stages, revealed that there is substantial sex-dependent gene expression, which predates even embryo implantation (Richardson et al., 2023). These sex-specific genes encode proteins with regulatory roles in sex-specific cellular development (e.g., protein–protein interaction networks, including epigenetic factors). During these early stages of embryogenesis, males show delays in DNA and RNA-related processes compared to delays in cell organization and cell compartmentalization seen in females. This theoretically sets the stage for sex-biased transcriptional and molecular differences in neurulation and neural stem cell development. Nonetheless, developmental programs between mice and humans differ, a fact that extends to the level of maternal transcripts; specifically, in humans, maternal programs are active until zygotic genome activation. Thus, translation of results from experimental models to humans must be done with caution.

In a study mapping lineage- and sex-specific gene activation during human embryonic development, Petropoulos et al. (2016) performed single-cell RNA-seq on human pre-implantation embryos (embryonic days 3–7), revealing that embryonic cell populations forming the trophectoderm, epiblast, and primitive endoderm initiate lineage-specific gene expression concurrently with forming the blastocyst. During this process, sex- specific X transcriptional dynamics diverge between male and female embryos. Specifically, in females, the three cell lineages forming the blastocyst achieve dosage compensation of X chromosome RNA levels prior to implantation. During the process of X chromosome dosage compensation, female pre-implantation embryonic cells maintain biallelic expression of *XIST* and X-linked genes. X dosage compensation starts on embryonic days 4–5. By embryonic day 7, it reaches 70–85% in the three layers. Moreover, during the pre-implantation period, sex differences are present between female and male cells with transcriptional differences in both X and autosomal gene expression. Interestingly, these sex-based differences are evident even before the initiation of the sex-determination program, as evidenced by the lack of *SRY* expression.

Taken together, the data presented suggest that sexual differentiation commences at fertilization, with sex-chromosome transcriptional and epigenetic factors controlling autosome expression to drive sex-specific lineage specification and sexually dimorphic features throughout life (Engel, 2018; Deegan and Engel, 2019).

### 2.2 Beyond sex-biased gene expression: long non-coding RNAs and alternative splicing

Genomic activity is not static but highly dynamic, with diversity in transcriptional activity playing a central role in sex-related biological processes, including sex-biased neurodevelopmental cascades that influence the manifestation of neurodevelopmental, neuropsychiatric, neurodegenerative, and cancer-related disorders (Ray et al., 2023; Mayne et al., 2016; Ober et al., 2008; Faustino and Cooper, 2003). Long non-coding RNAs (lncRNAs) constitute an important layer of transcriptional regulation and are increasingly recognized for their role in regulating higher-order brain functions (Stroud et al., 2021; Wang and Cooper, 2007).

During the earliest stages of embryogenesis, genomic activity is initially governed by maternally deposited transcripts until zygotic genome activation, after which the embryonic genome assumes control of morphogenesis. A recent study by the Larschan laboratory using *Drosophila melanogaster* embryos demonstrated that sex-specific differences in transcript diversity are present even in the earliest stages of development (Ray et al., 2023). These differences are driven by the maternally deposited pioneer factor CLAMP (chromatin-linked adapter for MSL proteins).

This study also showed that sex-specifically spliced isoforms are regulated by CLAMP during the first hours of embryogenesis, both before and after zygotic genome activation. Importantly, these findings provided the first evidence that sex-specific transcript diversity emerges well before zygotic genome activation. This process occurs concurrently with embryogenesis and is influenced by the maternal environment through CLAMP-mediated regulation of both zygotic genome activation and RNA transcript processing during zygotic development. Furthermore, CLAMP has also been shown to control neural stem cell proliferation and later synaptic circuit formation, suggesting that early-acting, sex-specific factors may switch their mode of action as neural development and differentiation proceed (Kentro et al., 2025; Tsiarli et al., 2025).

The presented findings are consistent with observations in other species, including humans, where early sex-biased gene expression has been detected near the onset of zygotic genome activation, alongside extensive chromatin landscape remodeling (Lowe et al., 2015). Together, these studies reveal an additional layer of regulatory control underlying early genomic activity and sex-specific developmental programming, which initiates upon conception and well before sex-specific hormonal influences.

Beyond the mechanisms discussed above, additional epigenetic mechanisms are also likely to contribute to sex-biased gene expression during neural development. DNA methylation, histone modifications, ATP-dependent chromatin remodeling, and chromatin accessibility are major regulators of transcriptional programs that govern neural progenitor maintenance, lineage specification, and neuronal differentiation. Although these chromatin-based mechanisms are increasingly recognized as important regulators of sexual differentiation of the brain, direct evidence demonstrating their role in sex chromosome-driven neurogenesis before gonadal differentiation remains limited. This represents an important avenue for future investigation using integrated single-cell transcriptomic and epigenomic approaches (Park et al., 2022; Kundakovic and Tickerhoof, 2024; Lafta et al., 2024).

## 3 Sex-differences following body plan establishment and initiation of nervous system development

In humans, embryo implantation takes place around days 19–22 of a typical 28-day menstrual cycle (Bull et al., 2019). Neurulation, the process by which the nervous system develops, initiates in the ectoderm following the formation of the three embryonic layers, i.e., gastrulation (Hill, 2007). The development of the human central nervous system is a complex process that initiates from approximately the fourth week of gestation and continues well beyond birth, with maturation of some brain regions extending into the third decade of life (Silbereis et al., 2016; Mousley et al., 2025). Furthermore, the central nervous system is one of the first organs to develop, and does so at an astonishingly fast pace, especially in the first years of postnatal life (Gogtay et al., 2004).

### 3.1 Sex-biased mechanisms during neural development

#### 3.1.1 Embryonic neural stem cells and early neural development

The X chromosome is enriched for neuronal genes, making it particularly important in neurodevelopment and in the sex bias observed in various neurodevelopmental disorders due to the process of X-inactivation (Lyon, 1961; Leblond et al., 2021). Specifically, compared to autosomal genes, X-linked genes have higher brain expression. Moreover, a higher proportion of X-linked genes (~sixfold higher on the X chromosome compared to autosomes) are associated with mutations leading to mental disability (Zechner et al., 2001; Nguyen and Disteche, 2006), which may be causal to the observation that mental disabilities are more common in males than females, by approximately 30%. Thus, X chromosome dynamics are key for the development and functions of the nervous system (Skuse, 2005; Disteche, 2006; Mallard et al., 2021; Raznahan and Disteche, 2021; Cabrera Zapata et al., 2022).

Due to the enrichment of neuronal-associated genes on the X-chromosome, most research has been directed into understanding the role of X-linked genes in brain development. This resulted in less studies investigating the Y-chromosome involvement in male brain development. Nonetheless, available data have pointed to the fact that both sex chromosomes have distinct roles in neuronal differentiation processes, eventually contributing to the manifestation of sex biases during human brain development and postnatally. In an important study addressing this topic, Pottmeier et al. (2024) used both male and female human embryonic stem cell lines (e.g., hESCs, WA14, and WA09, respectively) and examined genome-wide gene expression during *in vitro* neuronal differentiation over 37 days. The study revealed that hESCs contain inherent sex differences, as sex-biased gene expression is present from day 0 in undifferentiated hESCs and becomes most pronounced as the course of neural differentiation progresses. Among the sex-biased, differentially expressed genes are transcripts involved in neurodevelopment, suggesting that sex influences the differentiation trajectory. Interestingly, the male transcriptome exhibits the greatest contribution to differential gene expression, involving both Y chromosome and autosomal transcripts. Amongst the sex-differentially expressed transcripts are the Y-linked demethylases UTY (paralog of the X-linked UTX/KDM6A) and KDM5D, which were overexpressed during neuron development, confirming previous results in neural stem cells and mouse models (Walport et al., 2014; Shpargel et al., 2012; Rock et al., 2023).

#### 3.1.2 Forebrain development

Sex-biased transcriptional programs have also been identified during human fetal brain development. The large study by O’Brien et al. (2018) analyzed 120 prenatal human whole brain samples between 12 and 19 gestational weeks, corresponding to the second trimester of pregnancy, for sex-biased gene expression. Their analysis revealed more than 2,000 sex-biased genes, with male-biased genes enriched for cell cycle-related functions and predominantly expressed in neural progenitor populations. In contrast, female-biased genes displayed a more mature and lineage-committed transcriptional profile, with enrichment in synaptic signaling pathways and expression in Cajal–Retzius cells and glial populations.

Benoit-Pilven et al. (2025) explored the effects of sex on forebrain development in 266 RNA-seq samples (130 samples from 37 female individuals and 136 samples from 35 male individuals) from the first and early second trimesters, 5–17 weeks post-conception. Their study showed an abundance of sex-biased gene expression during forebrain development, with 58.5% of all sex-biased genes being in males, similar to previous reports (O’Brien et al., 2018). Furthermore, comparison of prenatal and adult forebrain data revealed that 1,000s of genes show stage-specific and/or shared sex bias, with prenatal sex-biased genes enriched for neuronal functions and expressed in neurons and neural stem cells. A substantial subset has continued sex bias across life, with similar direction and magnitude, indicating that many molecular sex differences are established prenatally and persist.

Collectively, these results reveal yet again that sex-biases in gene expression appear to be present from the very early stages, if not from the beginning, of embryonic development and manifest in a more pronounced way as neurogenesis progresses. Beyond their implications for sex-biased susceptibility to neurodevelopmental and neuropsychiatric disorders, these findings may also extend to other brain-related pathologies, including brain cancers, where marked sex differences are observed. For example, glioblastoma occurs more frequently in males than in females (Carrano et al., 2021; Pinares-Garcia et al., 2018), suggesting that sex-specific developmental and transcriptional programs may contribute to differential disease vulnerability later in life.

### 3.2 Sex hormone effects and brain architecture

At approximately the sixth week of human gestation (GW), the nascent, undifferentiated gonad initiates sexual differentiation under the influence of sex-specific transcriptional and molecular signaling. In male (46, XY) embryos, the Y chromosome gene *SRY* (Sex-determining Region Y) will guide gonad differentiation into testes (McElreavey et al., 1993). In female (46, XX) embryos, the absence of SRY signaling leads to ovarian formation. Testosterone production reaches a maximum between the 14th and 18th GW (Del Valle et al., 2017). Developing ovaries have limited steroidogenic activity; thus, female fetal estradiol production is low (Vaskivuo et al., 2005). Testosterone, along with other sex steroids that have differential expression in male versus female fetuses, plays a central role in brain sex differentiation. Thus, sex differences in brain development are the result of collective interactions between different sex-biased factors, mainly of the sex chromosomes and sex hormones, acting at critical developmental windows during the fetal life (Khan et al., 2026; Khan et al., 2024; Khramtsova et al., 2019; McCarthy and Arnold, 2011).

The activation of sex hormone signaling (androgens, estrogens, and progesterone) coincides with a surge in progenitor proliferation, neuronal migration, and the integration of nascent neurons into the cortical circuit, which, in combination with the established sex-genetic program, promotes the structuring of the cerebral cortex and drives sexual brain differentiation (Terrin et al., 2023). Activation of sex steroid transcriptional regulation networks results in neural network organization through synaptogenesis, neurite outgrowth, neuronal arborization, and myelination (Peper et al., 2011). In combination with sex chromosome transcriptional activity in the developing forebrain, steroid transcription factor binding sites are enriched within the sex-biased genes, adding an extra layer of control to the development and sexual differentiation of the brain. This change is more pronounced in the developing male forebrain (2nd trimester), with enrichment in 74 transcription factor binding sites that include the Androgen receptor, Estrogen Receptor 1 and their interactors such as *FOXA1*, *FOXP1,* and *JUN*. In the forebrains of female embryos, eight transcription factor binding sites were found to be upregulated in the dataset of sex-biased genes (Benoit-Pilven et al., 2025).

By 18 weeks of gestation, the brain has matured enough that structural brain differences become more evident (Studholme et al., 2020; Hostalet et al., 2025), and the lissencephalic human cortex transforms into a complex structure composed of gyri and sulci (Lohmann et al., 2008). The sulcation process has a sex-specific, temporary sequence that initiates on the 9th week of gestation with the formation of the primary sulci (Im, 2021; Habas et al., 2012). For example, in female fetuses, sulci of the right superior temporal sulcus emerge earlier compared to male fetuses (Yun et al., 2022). Recent results revealed that in addition to time differences in cortical architecture sequence, the number of sulcal pits in both hemispheres is sex-biased. Specifically, the study showed that males have a higher number of sulcal pits in both hemispheres compared to females (Hostalet et al., 2025). These results align with documented sex differences in neuroanatomy, which mainly focused on postnatal brain structural differences between the two sexes. These studies have shown that brain volume is on average larger in males than females, while cortical gray matter density is higher in females (Pavlinek et al., 2024; De Bellis et al., 2001; Ritchie et al., 2018; Luders et al., 2005). Khan et al. (2026) showed that prenatal brain growth rates also have a sex bias: in a prenatal MRI data sample ranging from 21–45 GW, male fetuses showed greater volumetric increases with age compared to females, adding to a continuum of sex differences in brain development and function that initiate in the womb and extend throughout postnatal life.

Overall, sex differences in brain structure and function begin *in utero* through the actions of sex chromosomes and hormone-dependent gene regulation, acting independently or in combination during critical prenatal developmental windows that shape brain development and function. Some of these mechanisms continue to influence neurodevelopment and function beyond birth.

## 4 Sex-biases in brain health and links to disease

As discussed throughout this review, sex influences brain development from the earliest stages of embryogenesis. These early sex-biased developmental programs shape postnatal neuronal circuitry and cognitive function (Mallard et al., 2021; Kostovic et al., 2019), contributing to sex differences in the manifestation of neurodevelopmental, neurological, and neuropsychiatric disorders (Jiang et al., 2025; Marchetto et al., 2017).

Sex bias in neurodevelopmental disorders is well documented, with females being affected less often and less severely than males (Pearse II and Young-Pearse, 2019), due to the fact that neurodevelopmental disorder-associated genes are overrepresented on the X chromosome (Leblond et al., 2021). In males, the majority of X-linked genes are hemizygous, i.e., males only carry the allele inherited from the mother. In contrast, random XCI in female cells results in a mosaic expression of alleles from the two X chromosomes in female tissues, which substantially influences the phenotype development of X-linked gene defects (Giovenino et al., 2023; Jiang et al., 2025).

Beyond the mosaicism and randomness associated with female X-inactivation, the reactivation of silenced X-linked genes has emerged as an additional factor contributing to the sex biased neural circuit development, function, and manifestation of neurodevelopmental disorders. In human induced pluripotent stem cells undergoing neural differentiation induction as organoids, genes on the inactive female X chromosome are reactivated and then silenced in a time-dependent manner associated with the progression of neuronal differentiation. These results are supported by single-cell RNA sequencing data from human embryo spinal cord. Specifically, the inactive X has emerged as a dynamic player in transcriptional regulation with time- and cell-type specific gene activation, with results showing reactivation of X genes is associated with neurodevelopmental disorders (Bertin et al., 2026). In studies investigating the postnatal effects of X inactivation and parental origin in mice, inactivation of the paternal X chromosome, leaving only the maternal X transcriptionally active, was associated with cognitive decline and brain aging phenotypes in female offspring (Abdulai-Saiku et al., 2025).

Cortical network architecture and neuronal layering are critical determinants of the development of neurodevelopmental and other brain disorders (Toledo et al., 2022). Cortical layer 2/3 pyramidal neurons are key mediators of signal integration in cognitive processes and have been implicated in the pathogenesis of neurodevelopmental disorders, epilepsy, and intellectual disability (Barington et al., 2018; Hoischen et al., 2014). Transcriptomic analysis of L2/3 neurons revealed a critical, time-restricted, sex-biased window of gene expression at postnatal day 4 in mice (Marcos-Grañeda et al., 2026), corresponding to approximately gestational week 24 in human development. Furthermore, male mice were more susceptible to the disruption of *Cux1* and *Cux2*, key transcription factor determinants of L2/3 neurons, with effects on sex-specific transcription patterns (Cottam et al., 2025). Although direct translation to humans remains to be explored, these findings may provide insight into sex-specific vulnerabilities in age-related cognitive decline and neurodegenerative diseases (Ziemka-Nalecz et al., 2023).

In addition to sex bias due to sex chromosomes, there have been documented sex differences in brain autosomal gene expression regulation, which, in turn, can contribute to the development of neurodevelopmental disorders and other brain-related conditions. Toledo et al. (2022) used a computational approach that included co-expression and functional enrichment analysis on published RNA-seq data from the midgestational fetal brain tissues (12–19 gestation weeks). The results showed sex biases in different biological processes, including cell metabolic profiles, metabolic functions of mitochondria in energy metabolism, apoptosis regulation, and steroid hormone production, with male cells showing higher proliferation markers, whereas female cells exhibited an earlier shift toward an oxidative metabolic profile associated with neuronal differentiation. Overall, males show greater metabolic sensitivity, which has been associated with the development of neurodevelopmental disorders (da Silveira Paulsen et al., 2012; Saudubray and Garcia-Cazorla, 2018; Garone et al., 2024).

## 5 Discussion

Sex differences in brain development and function have been extensively studied in neurodevelopmental and medical research. Traditionally, the majority of studies have focused on differences arising from gonadal differentiation and subsequent sex hormone signaling. However, these mechanisms alone do not fully explain the sexually dimorphic manifestations observed in neurodevelopmental, neuropsychiatric, and neurological disorders.

As highlighted throughout this review, the emergence of omics technologies, computational approaches, experimental model systems, and large-scale human datasets has provided deeper insights into the molecular and cellular mechanisms underlying sex-specific neural development. Increasing evidence indicates that the developing embryo exhibits sex-specific features during the pre-implantation period, well before the onset of gonadal formation and hormone-dependent regulation, in an interplay of sex chromosome and autosome transcriptional activity, lnRNAs and other regulatory factors (Pottmeier et al., 2024; Abdulai-Saiku et al., 2025; Ray et al., 2023; Kentro et al., 2025; Tsiarli et al., 2025). These findings suggest that some mechanisms influencing brain development and brain health postnatally may originate in the earliest stages of embryogenesis, potentially beginning at conception (Shi et al., 2025; Bermejo-Alvarez et al., 2011). Therefore, it is becoming clear that the field needs to invest in a more granular level of investigating sex-based differences in brain development and susceptibility to disease, especially at the very early stages of embryogenesis. Supporting this approach is single-cell evidence from the adult human cerebral cortex showing that sex has distinct effects on different cortical regions and cell types (DeCasien et al., 2025), further supporting a better integration of experimental approaches and models, including human data, to understand the early developmental mechanisms of sex bias in the pathophysiology of brain-related disorders in males and females.

**Figure 1:**
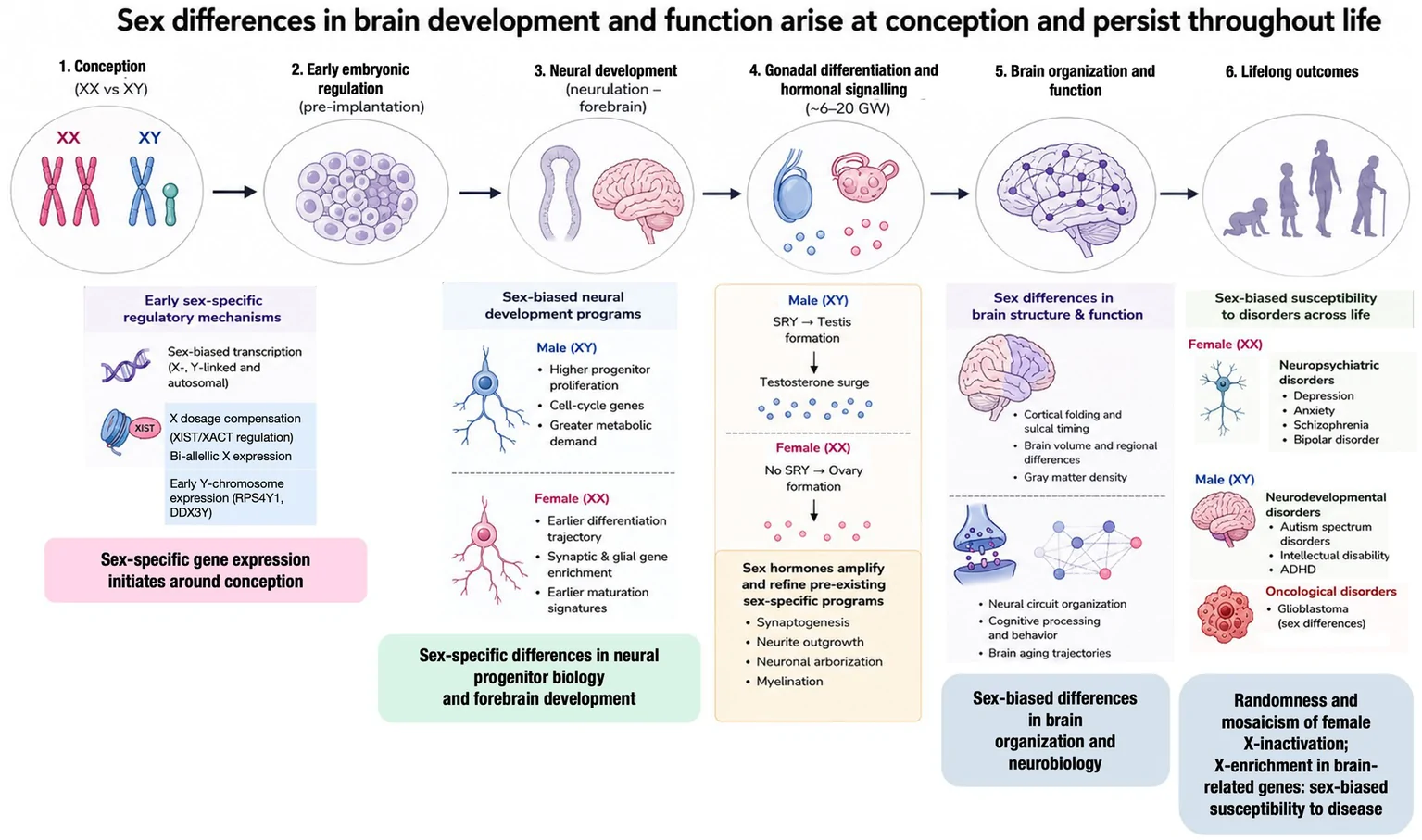
Sex differences in brain development and function arise at conception and persist throughout life. Sex chromosomes play an integral role in establishing early sex-specific differences from the very early stages of conception, which are subsequently amplified and refined by sex hormone signaling during embryonic development. Together, these mechanisms shape brain organization, function, and sex-biased susceptibility to disease across the lifespan.

Together, the evidence reviewed here supports a developmental framework in which sex-specific molecular programs emerge before gonadal differentiation and continue to influence neural development, brain organization, and disease susceptibility throughout life (Figure 1). Future studies integrating single-cell genomics, developmental neurobiology, and human and experimental model systems are essential to understanding how early sex-biased regulatory programs contribute to normal brain function and neurological disease.
